# Bis[2-(1*H*-1,2,4-triazol-1-yl-κ*N* ^2^)-1,10-phenanthroline-κ^2^ *N*,*N*′]cadmium(II) bis­(perchlorate)

**DOI:** 10.1107/S1600536809038768

**Published:** 2009-10-03

**Authors:** Hong Liang Li

**Affiliations:** a Department of Chemistry, Dezhou University, Dezhou 253023, People’s Republic of China

## Abstract

In the title complex, [Cd(C_14_H_9_N_5_)_2_](ClO_4_)_2_, the Cd^II^ ion is coordinated by two tridentate 2-(1*H*-1,2,4-triazol-1-yl)-1,10-phenanthroline ligands in a distorted octa­hedral CdN_6_ environment. In both 2-(1*H*-1,2,4-triazol-1-yl)-1,10-phenanthroline ligands, the 1,2,4-triazolyl ring and the 1,10-phenanthroline ring system are essentially coplanar [maximun deviations of 0.136 (7) and 0.273 (5) Å, respectively]. The dihedral angle between the mean planes of the ligands is 89.65 (4)°. In the crystal structure, there is a weak π–π stacking inter­action between a pyridine ring and a symmetry-related benzene ring with a centroid–centroid distance of 3.772 (3) Å.

## Related literature

For related structures, see: Li (2008 ▶); Liu *et al.* (2008 ▶).
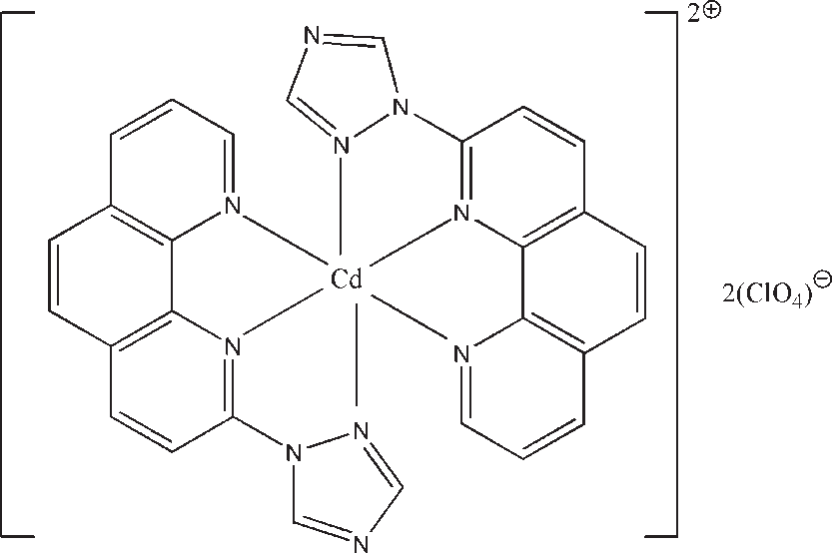


## Experimental

### 

#### Crystal data

[Cd(C_14_H_9_N_5_)_2_](ClO_4_)_2_
*M* *_r_* = 805.82
Monoclinic, 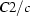

*a* = 16.894 (3) Å
*b* = 26.153 (5) Å
*c* = 15.574 (3) Å
β = 118.482 (2)°
*V* = 6048 (2) Å^3^
*Z* = 8
Mo *K*α radiation
μ = 0.97 mm^−1^
*T* = 298 K
0.39 × 0.34 × 0.29 mm

#### Data collection

Bruker SMART APEX CCD diffractometer
Absorption correction: multi-scan (*SADABS*; Sheldrick, 1996 ▶) *T*_min_ = 0.704, *T*_max_ = 0.766
17197 measured reflections
6543 independent reflections
4596 reflections with *I* > 2σ(*I*)
*R*_int_ = 0.042

#### Refinement

*R*[*F* ^2^ > 2σ(*F* ^2^)] = 0.054
*wR*(*F* ^2^) = 0.162
*S* = 1.08
6543 reflections
442 parameters
H-atom parameters constrained
Δρ_max_ = 1.44 e Å^−3^
Δρ_min_ = −0.47 e Å^−3^

### 

Data collection: *SMART* (Bruker, 1997 ▶); cell refinement: *SAINT* (Bruker, 1997 ▶); data reduction: *SAINT*; program(s) used to solve structure: *SHELXTL* (Sheldrick, 2008 ▶); program(s) used to refine structure: *SHELXTL*; molecular graphics: *SHELXTL*; software used to prepare material for publication: *SHELXTL*.

## Supplementary Material

Crystal structure: contains datablocks I, global. DOI: 10.1107/S1600536809038768/lh2911sup1.cif

Structure factors: contains datablocks I. DOI: 10.1107/S1600536809038768/lh2911Isup2.hkl

Additional supplementary materials:  crystallographic information; 3D view; checkCIF report

## Figures and Tables

**Fig. 1.**
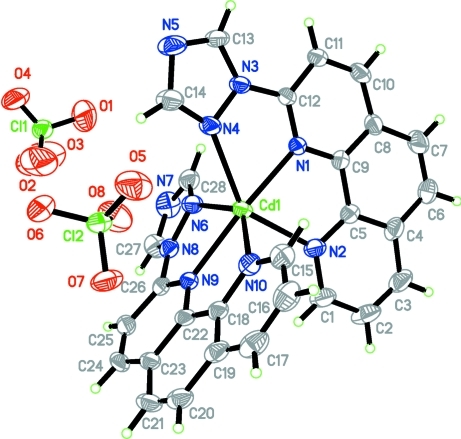
The asymmetric unit of the title compound with the atom-numbering scheme and displacement ellipsoids shown at the 30% probability level

## supplementary crystallographic information

### Comment

Derivatives of 1,10-phenanthroline play an important role in modern coordination chemistry (see e.g. Li, 2008; Liu *et al.* 2008), but until now the crystal structure of complexes with 2-(1*H*-1,2,4-triazol-1-yl)-1,10-phenanthroline as a ligand have not been reported. The crystal structure of the title complex is reported herein.

The asymmetric unit of the title complex is shown in Fig. 1. The Cd^II^ ion is coordinated in a distorted octahedral environment. In both 2-(1*H*-1,2,4-triazol-1-yl)-1,10-phenanthroline ligands, the 1,2,4-triazolyl ring and the 1,10-phenanthroline ring system are essentially co-planar within 0.0657 Å (for the ligand containing atom N1) and 0.1073 Å (for the ligand containing atom N6) with a maximun deviations of 0.136 (7) Å and 0.273 (5) Å for atom C6 and atom C27, respectively, and the dihedral angle between the two planes is 89.65 (4)°. In the crystal structure, there is a weak π–π stacking interaction involving a symmetry pyridine ring and symmetry related benzene ring, with the relevant distances being *Cg*1···*Cg*2^i^ = 3.772 (3) Å and *Cg*1···*Cg*2^i^_perp_ = 3.517 Å [symmetry code: (i) 1 - *x*, -*y*, 1 - *z*; *Cg*1 and *Cg*2 are the centroids of C1—C5/N2 ring and C4—C9 ring].

### Experimental

5 ml H_2_O solution of hydrated cadmium perchlorate (0.0535 g, 0.128 mmol) was added into 10 ml methanol solution of 2-(1*H*-1,2,4-triazol-1-yl)-1,10-phenanthroline (0.0631 g, 0.255 mmol) and the mixed soluton was stirred for a few minutes. Yellow single crystals were obtained after the filtrate was allowed to stand at room temperature for two weeks.

### Refinement

All H atoms were placed in calculated positions and refined as riding with C—H = 0.93Å and *U*_iso_ = 1.2*U*_eq_(C).

### Crystal data

**Table:**

[Cd(C_14_H_9_N_5_)_2_](ClO_4_)_2_	*F*(000) = 3216
*M**_r_* = 805.82	*D*_x_ = 1.770 Mg m^−^^3^
Monoclinic, *C*2/*c*	Mo *K*α radiation, λ = 0.71073 Å
Hall symbol: -C 2yc	Cell parameters from 6564 reflections
*a* = 16.894 (3) Å	θ = 2.6–27.7°
*b* = 26.153 (5) Å	µ = 0.97 mm^−^^1^
*c* = 15.574 (3) Å	*T* = 298 K
β = 118.482 (2)°	Block, yellow
*V* = 6048 (2) Å^3^	0.39 × 0.34 × 0.29 mm
*Z* = 8	

### Data collection

**Table:**

Bruker SMART APEX CCD diffractometer	6543 independent reflections
Radiation source: fine-focus sealed tube	4596 reflections with *I* > 2σ(*I*)
graphite	*R*_int_ = 0.042
φ and ω scans	θ_max_ = 27.0°, θ_min_ = 1.6°
Absorption correction: multi-scan (*SADABS*; Sheldrick, 1996)	*h* = −21→21
*T*_min_ = 0.704, *T*_max_ = 0.766	*k* = −28→33
17197 measured reflections	*l* = −19→19

### Refinement

**Table:**

Refinement on *F*^2^	Primary atom site location: structure-invariant direct methods
Least-squares matrix: full	Secondary atom site location: difference Fourier map
*R*[*F*^2^ > 2σ(*F*^2^)] = 0.054	Hydrogen site location: inferred from neighbouring sites
*wR*(*F*^2^) = 0.162	H-atom parameters constrained
*S* = 1.08	*w* = 1/[σ^2^(*F*_o_^2^) + (0.0899*P*)^2^ + 4.5706*P*] where *P* = (*F*_o_^2^ + 2*F*_c_^2^)/3
6543 reflections	(Δ/σ)_max_ = 0.008
442 parameters	Δρ_max_ = 1.44 e Å^−^^3^
0 restraints	Δρ_min_ = −0.47 e Å^−^^3^

### Special details

**Table:**

Geometry. All e.s.d.'s (except the e.s.d. in the dihedral angle between two l.s. planes) are estimated using the full covariance matrix. The cell e.s.d.'s are taken into account individually in the estimation of e.s.d.'s in distances, angles and torsion angles; correlations between e.s.d.'s in cell parameters are only used when they are defined by crystal symmetry. An approximate (isotropic) treatment of cell e.s.d.'s is used for estimating e.s.d.'s involving l.s. planes.
Refinement. Refinement of *F*^2^ against ALL reflections. The weighted *R*-factor *wR* and goodness of fit *S* are based on *F*^2^, conventional *R*-factors *R* are based on *F*, with *F* set to zero for negative *F*^2^. The threshold expression of *F*^2^ > σ(*F*^2^) is used only for calculating *R*-factors(gt) *etc*. and is not relevant to the choice of reflections for refinement. *R*-factors based on *F*^2^ are statistically about twice as large as those based on *F*, and *R*- factors based on ALL data will be even larger.

### Fractional atomic coordinates and isotropic or equivalent isotropic displacement parameters (Å^2^)

**Table:**

	*x*	*y*	*z*	*U*_iso_*/*U*_eq_	
C1	0.5127 (3)	0.1182 (2)	0.4227 (5)	0.0694 (16)	
H1	0.5150	0.1532	0.4338	0.083*	
C2	0.5935 (4)	0.0909 (3)	0.4576 (5)	0.0799 (19)	
H2	0.6483	0.1079	0.4915	0.096*	
C3	0.5919 (3)	0.0404 (2)	0.4420 (5)	0.0727 (17)	
H3	0.6456	0.0224	0.4642	0.087*	
C4	0.5090 (3)	0.0145 (2)	0.3922 (4)	0.0592 (13)	
C5	0.4305 (3)	0.04410 (17)	0.3603 (4)	0.0475 (11)	
C6	0.5000 (4)	−0.0394 (2)	0.3739 (5)	0.0759 (18)	
H6	0.5516	−0.0588	0.3913	0.091*	
C7	0.4194 (4)	−0.0627 (2)	0.3325 (5)	0.0731 (17)	
H7	0.4163	−0.0980	0.3247	0.088*	
C8	0.3382 (3)	−0.03361 (17)	0.3001 (4)	0.0486 (11)	
C9	0.3439 (3)	0.01938 (17)	0.3114 (3)	0.0427 (10)	
C10	0.2515 (4)	−0.05463 (18)	0.2560 (4)	0.0586 (13)	
H10	0.2449	−0.0899	0.2496	0.070*	
C11	0.1767 (3)	−0.02478 (17)	0.2222 (4)	0.0516 (12)	
H11	0.1190	−0.0387	0.1916	0.062*	
C12	0.1911 (3)	0.02789 (15)	0.2361 (3)	0.0391 (9)	
C13	0.0294 (3)	0.05598 (18)	0.1521 (4)	0.0597 (14)	
H13	0.0013	0.0243	0.1328	0.072*	
C14	0.0545 (4)	0.1336 (2)	0.1739 (5)	0.082 (2)	
H14	0.0445	0.1686	0.1719	0.099*	
C15	0.3177 (4)	0.1891 (2)	0.1320 (4)	0.0642 (14)	
H15	0.2954	0.1584	0.0992	0.077*	
C16	0.3438 (4)	0.2265 (3)	0.0876 (5)	0.0733 (16)	
H16	0.3413	0.2202	0.0276	0.088*	
C17	0.3730 (4)	0.2721 (2)	0.1323 (5)	0.0706 (16)	
H17	0.3902	0.2974	0.1026	0.085*	
C18	0.3535 (3)	0.24079 (16)	0.2655 (4)	0.0453 (11)	
C19	0.3776 (3)	0.28132 (19)	0.2236 (4)	0.0580 (14)	
C20	0.4052 (3)	0.3288 (2)	0.2756 (5)	0.0639 (15)	
H20	0.4212	0.3558	0.2480	0.077*	
C21	0.4088 (3)	0.33548 (18)	0.3603 (5)	0.0650 (16)	
H21	0.4277	0.3669	0.3916	0.078*	
C22	0.3583 (3)	0.24799 (16)	0.3577 (4)	0.0453 (11)	
C23	0.3839 (3)	0.29504 (16)	0.4074 (4)	0.0508 (12)	
C24	0.3833 (3)	0.29919 (18)	0.4969 (4)	0.0565 (14)	
H24	0.3966	0.3304	0.5293	0.068*	
C25	0.3637 (3)	0.25823 (19)	0.5368 (4)	0.0537 (12)	
H25	0.3648	0.2604	0.5969	0.064*	
C26	0.3419 (3)	0.21271 (17)	0.4836 (3)	0.0449 (11)	
C27	0.3297 (4)	0.1559 (2)	0.6069 (4)	0.0661 (14)	
H27	0.3577	0.1766	0.6619	0.079*	
C28	0.2613 (3)	0.0968 (2)	0.5116 (4)	0.0598 (13)	
H28	0.2302	0.0663	0.4879	0.072*	
Cd1	0.28772 (2)	0.135509 (12)	0.30429 (3)	0.04704 (15)	
Cl1	0.13943 (10)	0.06057 (5)	0.95941 (12)	0.0702 (4)	
Cl2	0.10794 (8)	0.28026 (5)	0.15282 (10)	0.0570 (3)	
N1	0.2704 (2)	0.04897 (13)	0.2791 (3)	0.0386 (8)	
N2	0.4328 (3)	0.09551 (14)	0.3743 (3)	0.0531 (10)	
N3	0.1183 (2)	0.06236 (13)	0.2033 (3)	0.0453 (9)	
N4	0.1360 (3)	0.11365 (14)	0.2175 (3)	0.0534 (10)	
N5	−0.0133 (3)	0.09957 (18)	0.1326 (5)	0.0868 (18)	
N6	0.2755 (3)	0.12904 (14)	0.4555 (3)	0.0503 (9)	
N7	0.2948 (4)	0.1110 (2)	0.6053 (4)	0.0757 (14)	
N8	0.3199 (2)	0.16812 (15)	0.5189 (3)	0.0462 (9)	
N9	0.3383 (2)	0.20762 (13)	0.3991 (3)	0.0412 (8)	
N10	0.3231 (3)	0.19511 (15)	0.2187 (3)	0.0501 (9)	
O1	0.1678 (5)	0.0166 (2)	0.9378 (7)	0.176 (3)	
O2	0.2009 (8)	0.0683 (6)	1.0566 (7)	0.278 (7)	
O3	0.1713 (6)	0.1011 (3)	0.9279 (7)	0.178 (3)	
O4	0.0568 (4)	0.0603 (3)	0.9342 (11)	0.310 (9)	
O5	0.0794 (4)	0.2506 (2)	0.0682 (4)	0.1056 (17)	
O6	0.0358 (3)	0.30873 (18)	0.1493 (4)	0.1030 (17)	
O7	0.1776 (3)	0.31420 (17)	0.1655 (4)	0.1020 (18)	
O8	0.1407 (4)	0.24674 (18)	0.2350 (3)	0.0993 (15)	

### Atomic displacement parameters (Å^2^)

**Table:**

	*U*^11^	*U*^22^	*U*^33^	*U*^12^	*U*^13^	*U*^23^
C1	0.042 (3)	0.059 (3)	0.094 (4)	−0.008 (2)	0.021 (3)	0.006 (3)
C2	0.038 (3)	0.087 (5)	0.097 (5)	−0.011 (3)	0.018 (3)	0.015 (4)
C3	0.038 (3)	0.078 (4)	0.096 (5)	0.008 (3)	0.028 (3)	0.010 (3)
C4	0.046 (3)	0.062 (3)	0.073 (4)	0.014 (2)	0.031 (3)	0.008 (3)
C5	0.040 (2)	0.047 (3)	0.055 (3)	0.0001 (19)	0.021 (2)	−0.001 (2)
C6	0.051 (3)	0.063 (4)	0.107 (5)	0.020 (3)	0.032 (3)	−0.006 (3)
C7	0.062 (3)	0.048 (3)	0.092 (4)	0.015 (3)	0.023 (3)	−0.009 (3)
C8	0.054 (3)	0.039 (2)	0.052 (3)	0.004 (2)	0.026 (2)	−0.005 (2)
C9	0.041 (2)	0.043 (2)	0.047 (2)	0.0028 (18)	0.023 (2)	−0.0007 (19)
C10	0.063 (3)	0.033 (2)	0.074 (4)	−0.003 (2)	0.028 (3)	−0.011 (2)
C11	0.045 (2)	0.037 (2)	0.068 (3)	−0.0058 (19)	0.023 (2)	−0.009 (2)
C12	0.039 (2)	0.037 (2)	0.039 (2)	−0.0017 (17)	0.0164 (19)	−0.0045 (17)
C13	0.037 (2)	0.039 (2)	0.082 (4)	−0.0066 (19)	0.011 (2)	−0.001 (2)
C14	0.043 (3)	0.043 (3)	0.115 (5)	0.005 (2)	0.001 (3)	0.000 (3)
C15	0.064 (3)	0.064 (3)	0.071 (4)	−0.002 (3)	0.038 (3)	−0.001 (3)
C16	0.069 (4)	0.094 (5)	0.068 (4)	0.007 (3)	0.041 (3)	0.012 (3)
C17	0.056 (3)	0.081 (4)	0.081 (4)	0.005 (3)	0.037 (3)	0.031 (3)
C18	0.0301 (19)	0.038 (2)	0.061 (3)	0.0026 (17)	0.017 (2)	0.005 (2)
C19	0.037 (2)	0.050 (3)	0.084 (4)	0.006 (2)	0.026 (3)	0.021 (3)
C20	0.042 (3)	0.047 (3)	0.094 (4)	0.000 (2)	0.026 (3)	0.020 (3)
C21	0.046 (3)	0.033 (2)	0.094 (4)	−0.006 (2)	0.015 (3)	0.002 (3)
C22	0.0260 (19)	0.035 (2)	0.063 (3)	0.0003 (16)	0.012 (2)	0.002 (2)
C23	0.029 (2)	0.034 (2)	0.072 (3)	0.0014 (17)	0.009 (2)	0.002 (2)
C24	0.039 (2)	0.043 (3)	0.065 (3)	0.0014 (19)	0.007 (2)	−0.020 (2)
C25	0.035 (2)	0.054 (3)	0.056 (3)	0.004 (2)	0.009 (2)	−0.013 (2)
C26	0.0277 (19)	0.047 (2)	0.052 (3)	0.0050 (17)	0.0134 (19)	−0.005 (2)
C27	0.072 (4)	0.067 (3)	0.053 (3)	0.002 (3)	0.024 (3)	−0.001 (3)
C28	0.053 (3)	0.053 (3)	0.074 (4)	0.006 (2)	0.030 (3)	0.007 (3)
Cd1	0.0425 (2)	0.0334 (2)	0.0606 (3)	−0.00736 (12)	0.02088 (17)	−0.00628 (14)
Cl1	0.0631 (8)	0.0591 (8)	0.0876 (11)	−0.0039 (6)	0.0353 (8)	0.0056 (7)
Cl2	0.0429 (6)	0.0549 (7)	0.0676 (8)	−0.0099 (5)	0.0218 (6)	−0.0054 (6)
N1	0.0362 (17)	0.0336 (17)	0.0435 (19)	0.0004 (14)	0.0170 (16)	−0.0021 (15)
N2	0.040 (2)	0.045 (2)	0.070 (3)	−0.0043 (16)	0.023 (2)	0.0012 (19)
N3	0.0385 (18)	0.0323 (18)	0.055 (2)	−0.0049 (14)	0.0139 (17)	−0.0016 (16)
N4	0.042 (2)	0.0306 (18)	0.073 (3)	−0.0002 (16)	0.015 (2)	−0.0007 (18)
N5	0.037 (2)	0.054 (3)	0.124 (4)	−0.001 (2)	0.002 (3)	0.001 (3)
N6	0.045 (2)	0.046 (2)	0.056 (2)	−0.0054 (16)	0.0210 (19)	−0.0009 (18)
N7	0.092 (4)	0.078 (3)	0.069 (3)	0.004 (3)	0.048 (3)	0.011 (3)
N8	0.0382 (19)	0.052 (2)	0.046 (2)	0.0004 (16)	0.0179 (17)	−0.0018 (18)
N9	0.0326 (17)	0.0335 (18)	0.053 (2)	0.0004 (14)	0.0171 (16)	−0.0003 (16)
N10	0.047 (2)	0.049 (2)	0.056 (2)	0.0022 (17)	0.0256 (19)	0.0061 (18)
O1	0.150 (6)	0.085 (4)	0.294 (10)	0.035 (4)	0.106 (6)	0.011 (5)
O2	0.228 (11)	0.47 (2)	0.156 (8)	−0.129 (12)	0.111 (8)	−0.059 (10)
O3	0.188 (7)	0.104 (5)	0.223 (8)	−0.025 (5)	0.083 (6)	0.050 (5)
O4	0.064 (4)	0.128 (6)	0.64 (2)	−0.014 (4)	0.089 (8)	−0.112 (9)
O5	0.125 (4)	0.123 (4)	0.086 (3)	−0.046 (3)	0.065 (3)	−0.039 (3)
O6	0.053 (2)	0.088 (3)	0.156 (5)	−0.001 (2)	0.040 (3)	−0.016 (3)
O7	0.052 (2)	0.072 (3)	0.172 (5)	−0.018 (2)	0.045 (3)	−0.001 (3)
O8	0.126 (4)	0.080 (3)	0.085 (3)	−0.011 (3)	0.045 (3)	0.005 (2)

### Geometric parameters (Å, °)

**Table:**

C1—N2	1.331 (6)	C18—C19	1.403 (6)
C1—C2	1.399 (8)	C18—C22	1.412 (7)
C1—H1	0.9300	C19—C20	1.434 (8)
C2—C3	1.343 (8)	C20—C21	1.304 (9)
C2—H2	0.9300	C20—H20	0.9300
C3—C4	1.409 (7)	C21—C23	1.458 (7)
C3—H3	0.9300	C21—H21	0.9300
C4—C5	1.406 (6)	C22—N9	1.362 (6)
C4—C6	1.430 (8)	C22—C23	1.407 (6)
C5—N2	1.359 (6)	C23—C24	1.403 (8)
C5—C9	1.440 (6)	C24—C25	1.356 (7)
C6—C7	1.343 (8)	C24—H24	0.9300
C6—H6	0.9300	C25—C26	1.396 (6)
C7—C8	1.435 (7)	C25—H25	0.9300
C7—H7	0.9300	C26—N9	1.294 (6)
C8—C9	1.395 (6)	C26—N8	1.413 (6)
C8—C10	1.400 (7)	C27—N7	1.310 (8)
C9—N1	1.340 (5)	C27—N8	1.338 (7)
C10—C11	1.359 (7)	C27—H27	0.9300
C10—H10	0.9300	C28—N6	1.316 (6)
C11—C12	1.398 (6)	C28—N7	1.341 (7)
C11—H11	0.9300	C28—H28	0.9300
C12—N1	1.300 (5)	Cd1—N1	2.292 (3)
C12—N3	1.410 (5)	Cd1—N9	2.295 (3)
C13—N5	1.305 (6)	Cd1—N10	2.307 (4)
C13—N3	1.333 (6)	Cd1—N4	2.327 (4)
C13—H13	0.9300	Cd1—N2	2.396 (4)
C14—N4	1.318 (7)	Cd1—N6	2.465 (4)
C14—N5	1.347 (7)	Cl1—O4	1.257 (7)
C14—H14	0.9300	Cl1—O1	1.349 (7)
C15—N10	1.319 (7)	Cl1—O3	1.380 (7)
C15—C16	1.385 (8)	Cl1—O2	1.382 (10)
C15—H15	0.9300	Cl2—O5	1.402 (5)
C16—C17	1.350 (8)	Cl2—O6	1.407 (5)
C16—H16	0.9300	Cl2—O7	1.410 (4)
C17—C19	1.408 (9)	Cl2—O8	1.428 (5)
C17—H17	0.9300	N3—N4	1.369 (5)
C18—N10	1.366 (6)	N6—N8	1.370 (5)
			
N2—C1—C2	122.1 (5)	C25—C24—C23	120.9 (4)
N2—C1—H1	119.0	C25—C24—H24	119.6
C2—C1—H1	119.0	C23—C24—H24	119.6
C3—C2—C1	120.0 (5)	C24—C25—C26	117.1 (5)
C3—C2—H2	120.0	C24—C25—H25	121.5
C1—C2—H2	120.0	C26—C25—H25	121.5
C2—C3—C4	120.1 (5)	N9—C26—C25	124.2 (5)
C2—C3—H3	119.9	N9—C26—N8	114.7 (4)
C4—C3—H3	119.9	C25—C26—N8	121.1 (5)
C5—C4—C3	116.9 (5)	N7—C27—N8	111.1 (5)
C5—C4—C6	118.7 (5)	N7—C27—H27	124.5
C3—C4—C6	124.4 (5)	N8—C27—H27	124.5
N2—C5—C4	122.5 (4)	N6—C28—N7	115.5 (5)
N2—C5—C9	118.3 (4)	N6—C28—H28	122.3
C4—C5—C9	119.2 (4)	N7—C28—H28	122.3
C7—C6—C4	122.0 (5)	N1—Cd1—N9	154.12 (13)
C7—C6—H6	119.0	N1—Cd1—N10	127.99 (14)
C4—C6—H6	119.0	N9—Cd1—N10	72.38 (14)
C6—C7—C8	120.6 (5)	N1—Cd1—N4	69.07 (12)
C6—C7—H7	119.7	N9—Cd1—N4	122.99 (13)
C8—C7—H7	119.7	N10—Cd1—N4	110.32 (14)
C9—C8—C10	116.4 (4)	N1—Cd1—N2	70.49 (12)
C9—C8—C7	119.1 (4)	N9—Cd1—N2	94.59 (13)
C10—C8—C7	124.5 (4)	N10—Cd1—N2	93.84 (14)
N1—C9—C8	122.0 (4)	N4—Cd1—N2	139.56 (13)
N1—C9—C5	117.7 (4)	N1—Cd1—N6	91.92 (12)
C8—C9—C5	120.3 (4)	N9—Cd1—N6	67.45 (13)
C11—C10—C8	121.7 (4)	N10—Cd1—N6	139.53 (14)
C11—C10—H10	119.1	N4—Cd1—N6	88.13 (15)
C8—C10—H10	119.1	N2—Cd1—N6	93.95 (14)
C10—C11—C12	116.4 (4)	O4—Cl1—O1	113.0 (5)
C10—C11—H11	121.8	O4—Cl1—O3	117.2 (7)
C12—C11—H11	121.8	O1—Cl1—O3	108.7 (6)
N1—C12—C11	124.0 (4)	O4—Cl1—O2	119.1 (9)
N1—C12—N3	115.0 (3)	O1—Cl1—O2	103.1 (8)
C11—C12—N3	121.1 (4)	O3—Cl1—O2	93.4 (6)
N5—C13—N3	111.7 (4)	O5—Cl2—O6	110.7 (4)
N5—C13—H13	124.1	O5—Cl2—O7	111.7 (3)
N3—C13—H13	124.1	O6—Cl2—O7	108.8 (3)
N4—C14—N5	115.3 (5)	O5—Cl2—O8	108.4 (3)
N4—C14—H14	122.4	O6—Cl2—O8	108.5 (4)
N5—C14—H14	122.4	O7—Cl2—O8	108.7 (3)
N10—C15—C16	123.0 (6)	C12—N1—C9	119.4 (4)
N10—C15—H15	118.5	C12—N1—Cd1	121.5 (3)
C16—C15—H15	118.5	C9—N1—Cd1	119.1 (3)
C17—C16—C15	119.4 (6)	C1—N2—C5	118.4 (4)
C17—C16—H16	120.3	C1—N2—Cd1	127.2 (4)
C15—C16—H16	120.3	C5—N2—Cd1	114.4 (3)
C16—C17—C19	120.2 (5)	C13—N3—N4	108.4 (3)
C16—C17—H17	119.9	C13—N3—C12	132.6 (4)
C19—C17—H17	119.9	N4—N3—C12	118.8 (3)
N10—C18—C19	122.1 (5)	C14—N4—N3	102.1 (4)
N10—C18—C22	118.9 (4)	C14—N4—Cd1	142.4 (3)
C19—C18—C22	118.9 (4)	N3—N4—Cd1	115.4 (3)
C18—C19—C17	116.8 (5)	C13—N5—C14	102.4 (4)
C18—C19—C20	118.7 (5)	C28—N6—N8	102.1 (4)
C17—C19—C20	124.5 (5)	C28—N6—Cd1	143.4 (4)
C21—C20—C19	122.2 (5)	N8—N6—Cd1	111.8 (3)
C21—C20—H20	118.9	C27—N7—C28	102.8 (5)
C19—C20—H20	118.9	C27—N8—N6	108.6 (4)
C20—C21—C23	121.8 (5)	C27—N8—C26	131.9 (4)
C20—C21—H21	119.1	N6—N8—C26	119.4 (4)
C23—C21—H21	119.1	C26—N9—C22	120.0 (4)
N9—C22—C23	119.9 (5)	C26—N9—Cd1	124.1 (3)
N9—C22—C18	118.2 (4)	C22—N9—Cd1	115.6 (3)
C23—C22—C18	121.9 (4)	C15—N10—C18	118.4 (4)
C24—C23—C22	117.9 (4)	C15—N10—Cd1	126.9 (4)
C24—C23—C21	125.7 (5)	C18—N10—Cd1	114.7 (3)
C22—C23—C21	116.4 (5)		
			
N2—C1—C2—C3	0.3 (11)	N4—Cd1—N2—C5	1.2 (5)
C1—C2—C3—C4	−1.2 (11)	N6—Cd1—N2—C5	92.9 (4)
C2—C3—C4—C5	0.3 (9)	N5—C13—N3—N4	−0.9 (7)
C2—C3—C4—C6	−178.5 (7)	N5—C13—N3—C12	−175.9 (5)
C3—C4—C5—N2	1.4 (8)	N1—C12—N3—C13	175.5 (5)
C6—C4—C5—N2	−179.7 (6)	C11—C12—N3—C13	−4.6 (8)
C3—C4—C5—C9	−178.3 (5)	N1—C12—N3—N4	0.9 (6)
C6—C4—C5—C9	0.6 (8)	C11—C12—N3—N4	−179.2 (5)
C5—C4—C6—C7	−3.9 (10)	N5—C14—N4—N3	−0.6 (8)
C3—C4—C6—C7	174.9 (7)	N5—C14—N4—Cd1	179.9 (5)
C4—C6—C7—C8	3.3 (11)	C13—N3—N4—C14	0.8 (6)
C6—C7—C8—C9	0.6 (9)	C12—N3—N4—C14	176.6 (5)
C6—C7—C8—C10	179.8 (6)	C13—N3—N4—Cd1	−179.4 (4)
C10—C8—C9—N1	−2.0 (7)	C12—N3—N4—Cd1	−3.6 (5)
C7—C8—C9—N1	177.2 (5)	N1—Cd1—N4—C14	−177.1 (8)
C10—C8—C9—C5	176.9 (5)	N9—Cd1—N4—C14	28.8 (8)
C7—C8—C9—C5	−3.8 (8)	N10—Cd1—N4—C14	−52.9 (8)
N2—C5—C9—N1	2.4 (7)	N2—Cd1—N4—C14	−175.9 (7)
C4—C5—C9—N1	−177.8 (4)	N6—Cd1—N4—C14	90.2 (8)
N2—C5—C9—C8	−176.5 (5)	N1—Cd1—N4—N3	3.4 (3)
C4—C5—C9—C8	3.2 (7)	N9—Cd1—N4—N3	−150.8 (3)
C9—C8—C10—C11	2.7 (8)	N10—Cd1—N4—N3	127.5 (3)
C7—C8—C10—C11	−176.5 (6)	N2—Cd1—N4—N3	4.5 (5)
C8—C10—C11—C12	−1.5 (8)	N6—Cd1—N4—N3	−89.4 (3)
C10—C11—C12—N1	−0.5 (8)	N3—C13—N5—C14	0.5 (8)
C10—C11—C12—N3	179.5 (5)	N4—C14—N5—C13	0.1 (10)
N10—C15—C16—C17	−2.7 (9)	N7—C28—N6—N8	−1.9 (6)
C15—C16—C17—C19	0.6 (9)	N7—C28—N6—Cd1	155.8 (4)
N10—C18—C19—C17	−2.9 (7)	N1—Cd1—N6—C28	−3.2 (6)
C22—C18—C19—C17	179.1 (4)	N9—Cd1—N6—C28	−167.1 (6)
N10—C18—C19—C20	177.4 (4)	N10—Cd1—N6—C28	−174.4 (5)
C22—C18—C19—C20	−0.7 (6)	N4—Cd1—N6—C28	65.8 (6)
C16—C17—C19—C18	2.0 (7)	N2—Cd1—N6—C28	−73.7 (6)
C16—C17—C19—C20	−178.2 (5)	N1—Cd1—N6—N8	153.3 (3)
C18—C19—C20—C21	0.1 (7)	N9—Cd1—N6—N8	−10.6 (3)
C17—C19—C20—C21	−179.7 (5)	N10—Cd1—N6—N8	−17.9 (4)
C19—C20—C21—C23	−0.6 (8)	N4—Cd1—N6—N8	−137.7 (3)
N10—C18—C22—N9	4.5 (6)	N2—Cd1—N6—N8	82.7 (3)
C19—C18—C22—N9	−177.3 (4)	N8—C27—N7—C28	−1.4 (7)
N10—C18—C22—C23	−176.2 (4)	N6—C28—N7—C27	2.1 (7)
C19—C18—C22—C23	1.9 (6)	N7—C27—N8—N6	0.3 (6)
N9—C22—C23—C24	−3.2 (6)	N7—C27—N8—C26	176.4 (5)
C18—C22—C23—C24	177.5 (4)	C28—N6—N8—C27	1.0 (5)
N9—C22—C23—C21	176.9 (4)	Cd1—N6—N8—C27	−165.0 (3)
C18—C22—C23—C21	−2.3 (6)	C28—N6—N8—C26	−175.7 (4)
C20—C21—C23—C24	−178.2 (5)	Cd1—N6—N8—C26	18.4 (4)
C20—C21—C23—C22	1.7 (7)	N9—C26—N8—C27	167.5 (5)
C22—C23—C24—C25	3.8 (6)	C25—C26—N8—C27	−13.6 (7)
C21—C23—C24—C25	−176.4 (4)	N9—C26—N8—N6	−16.7 (5)
C23—C24—C25—C26	−1.8 (6)	C25—C26—N8—N6	162.1 (4)
C24—C25—C26—N9	−0.8 (6)	C25—C26—N9—C22	1.3 (6)
C24—C25—C26—N8	−179.6 (4)	N8—C26—N9—C22	−179.8 (3)
C11—C12—N1—C9	1.2 (7)	C25—C26—N9—Cd1	−173.1 (3)
N3—C12—N1—C9	−178.9 (4)	N8—C26—N9—Cd1	5.7 (5)
C11—C12—N1—Cd1	−177.4 (4)	C23—C22—N9—C26	0.8 (6)
N3—C12—N1—Cd1	2.5 (5)	C18—C22—N9—C26	−180.0 (4)
C8—C9—N1—C12	0.2 (7)	C23—C22—N9—Cd1	175.7 (3)
C5—C9—N1—C12	−178.8 (4)	C18—C22—N9—Cd1	−5.1 (5)
C8—C9—N1—Cd1	178.8 (4)	N1—Cd1—N9—C26	−36.7 (5)
C5—C9—N1—Cd1	−0.2 (5)	N10—Cd1—N9—C26	177.7 (3)
N9—Cd1—N1—C12	119.9 (4)	N4—Cd1—N9—C26	74.5 (4)
N10—Cd1—N1—C12	−103.2 (3)	N2—Cd1—N9—C26	−89.7 (3)
N4—Cd1—N1—C12	−3.3 (3)	N6—Cd1—N9—C26	2.7 (3)
N2—Cd1—N1—C12	177.5 (4)	N1—Cd1—N9—C22	148.6 (3)
N6—Cd1—N1—C12	84.0 (3)	N10—Cd1—N9—C22	3.0 (3)
N9—Cd1—N1—C9	−58.7 (5)	N4—Cd1—N9—C22	−100.2 (3)
N10—Cd1—N1—C9	78.2 (4)	N2—Cd1—N9—C22	95.6 (3)
N4—Cd1—N1—C9	178.1 (4)	N6—Cd1—N9—C22	−172.0 (3)
N2—Cd1—N1—C9	−1.1 (3)	C16—C15—N10—C18	1.9 (8)
N6—Cd1—N1—C9	−94.6 (3)	C16—C15—N10—Cd1	−177.4 (4)
C2—C1—N2—C5	1.3 (9)	C19—C18—N10—C15	1.0 (6)
C2—C1—N2—Cd1	−177.7 (5)	C22—C18—N10—C15	179.0 (4)
C4—C5—N2—C1	−2.2 (8)	C19—C18—N10—Cd1	−179.7 (3)
C9—C5—N2—C1	177.5 (5)	C22—C18—N10—Cd1	−1.6 (5)
C4—C5—N2—Cd1	177.0 (4)	N1—Cd1—N10—C15	16.8 (5)
C9—C5—N2—Cd1	−3.3 (6)	N9—Cd1—N10—C15	178.6 (4)
N1—Cd1—N2—C1	−178.6 (5)	N4—Cd1—N10—C15	−62.0 (4)
N9—Cd1—N2—C1	−20.3 (5)	N2—Cd1—N10—C15	85.0 (4)
N10—Cd1—N2—C1	52.3 (5)	N6—Cd1—N10—C15	−174.4 (4)
N4—Cd1—N2—C1	−179.7 (5)	N1—Cd1—N10—C18	−162.5 (3)
N6—Cd1—N2—C1	−87.9 (5)	N9—Cd1—N10—C18	−0.7 (3)
N1—Cd1—N2—C5	2.3 (3)	N4—Cd1—N10—C18	118.7 (3)
N9—Cd1—N2—C5	160.6 (4)	N2—Cd1—N10—C18	−94.3 (3)
N10—Cd1—N2—C5	−126.8 (4)	N6—Cd1—N10—C18	6.3 (4)
